# Association between work time loss and quality of life in patients with Herpes Zoster: a pooled analysis of the MASTER studies

**DOI:** 10.1186/s12955-017-0588-x

**Published:** 2017-01-18

**Authors:** Emmanouil Rampakakis, Melissa Stutz, Kosuke Kawai, Tsen-Fang Tsai, Hee Jin Cheong, Jittima Dhitavat, Alejandro Ortiz-Covarrubias, Miguel Cashat-Cruz, Homero Monsanto, Kelly D. Johnson, John S. Sampalis, Camilo J. Acosta

**Affiliations:** 1 JSS Medical Research, 9400 Henri-Bourassa W, St-Laurent, QC H4S 1N8 Canada; 2 Clinical Research Center, Boston Children’s Hospital, Harvard Medical School, 300 Longwood Ave., Boston, MA 02115 USA; 3 Department of Dermatology, National Taiwan University Hospital, No. 7, Zhongshan South Road, Zhongzheng District, Taipei City, 100 Taiwan; 4 Division of Infectious diseases, Department of Internal Medicine, Korea University Guro Hospital, 145 Anam-ro, Seongbuk-gu, Seoul, South Korea; 5 Clinical Infectious Disease Research Unit, Department of Clinical Tropical Medicine, Faculty of Tropical Medicine, Mahidol University, 420/6 Ratchawithi Road, Ratchathewi, Bangkok, 10400 Thailand; 6 Hospital Civil de Guadalajara Fray Antonio Alcalde, Calle Coronel Calderón #777, El Retiro, 44280 Guadalajara, Jalisco Mexico; 7 Vaccines Latin America & the Caribbean, MSD Corp., Av. San Jerónimo 369 Piso 8, Col. La Otra Banda, Mexico City, CP 01090 Mexico; 8 Latin America Health Outcomes Research, MSD (I.A.) Corp., P.O. Box 3689, Carolina, PR 00984-3689 Puerto Rico; 9 Center for Observational and Real-World Evidence, Merck & Co., Inc., 2000 Galloping Hill Rd, Kenilworth, NJ 07033 USA; 10 McGill University, 845 Sherbrooke Street W, Montreal, QC H3A 0G4 Canada

**Keywords:** Herpes Zoster, Quality of life, Work, Observational study

## Abstract

**Background:**

Herpes zoster (HZ) has a significant negative effect on the productive work life of individuals, and has been shown to be responsible for cases of absenteeism, presenteeism and decreased work effectiveness. The aim of this study was to evaluate health utility scores and associated predictors in an actively employed population of Herpes Zoster (HZ) patients with and without work time loss (WTL).

**Methods:**

This was a pooled analysis of the prospective, observational MASTER cohort studies, conducted in 8 countries across North America, Latin America and Asia. A total of 428 HZ patients engaged in full or part time work were included. WTL, defined as missing ≥ 1 partial or full work day, and work effectiveness, reported on a scale of 0–100%, were evaluated with the Work and Productivity Questionnaire (WPQ). The Pearson product–moment correlation was used to assess the correlation between work effectiveness and HRQoL. Mixed models with repeated measures assessed the relationship between HZ-related WTL over a 6-month follow-up period, and HRQoL, as evaluated by the EQ-5D. Additional predictors of HRQoL were also identified.

**Results:**

Overall, 57.7% of respondents reported WTL. Mean (SD) percent work effectiveness of patients in the WTL group was significantly lower compared to non-WTL (NWTL) patients at baseline [50.3 (31.6) vs. 71.4 (27.8); *p* < 0.001]. Patients in the WTL group also reported lower health utility scores at baseline and overall than their NWTL counterparts, with WTL identified as an independent negative predictor of both the EQ-5D summary scores and the EQ-5D VAS (*p* < 0.001). Decrease in work effectiveness was negatively associated with HRQoL overall (*p* < 0.001). Predictors of lower HRQoL were worst Zoster Brief Pain Inventory (ZBPI) pain score, the presence of HZ complications and country income (predictor of EQ-5D VAS only).

**Conclusions:**

HZ adversely impacts the work and productive life of actively employed individuals. In turn, HZ-related reductions in work effectiveness and work time are associated with a negative effect on HRQoL.

**Electronic supplementary material:**

The online version of this article (doi:10.1186/s12955-017-0588-x) contains supplementary material, which is available to authorized users.

## Background

HZ or shingles is caused by the reactivation of the varicella zoster virus (VZV), for which primary infection manifests as chickenpox, or varicella. The estimated lifetime risk for the development of HZ is approximately 30% [1–3]. Rash onset, the typical clinical feature of HZ, is characterized by a unilateral, dermatomal rash with vesicular lesions that usually heal within 2–4 weeks [4]. Pain during this phase, which ranges from moderate-to-severe in the majority of patients [5], negatively impacts functional status and QoL with greater acute pain burden significantly associated with poorer physical role, social functioning, and greater emotional distress [5–8]. HZ has also been shown to be responsible for cases of absenteeism, presenteeism (defined as attending work while sick) and decreased work effectiveness [9–11], with combined work loss varying significantly by disease severity [10]. Consequently, HZ has a significant negative effect on the productive work life of individuals; consideration of this effect on work and productivity therefore contributes to the cost-effectiveness of HZ therapeutic interventions, specifically those aiming at preventing VZV reactivation.

However, the true cost-effectiveness of therapeutic interventions for HZ may be underestimated. This is emphasized by discrepancies in guidelines for cost-effectiveness analysis, which differ as to whether work time loss should be included in the numerator of the incremental cost-effectiveness ratio (ICER), or as an implicit consideration of health state valuations, which are contained in the denominator of the ICER. This is due to the fact that it is unclear whether or not people actually take into account the effect of disease on their ability to work, and the resulting lost wages, when evaluating health states [12–14]. The Panel on Cost-Effectiveness in Health and Medicine have nevertheless recently put forth revised recommendations advocating for the inclusion of these effects in the numerator in the ICER [15].

The MASTER (Monitoring and Assessing Shingles Through Education and Research) studies, were prospective cohort studies conducted in 8 countries [16–19], which assessed Herpes Zoster (HZ)-related burden of illness, including pain, health related quality of life (HRQoL), health care resource utilisation (HCRU), and associated cost. Using data from these studies, the aim of this pooled analysis was to examine the association of HZ-related work time loss, or HZ-related work productivity loss, with HRQoL, to evaluate whether or not patients with work time loss have lower health utility scores than those without, and to identify predictors of HRQoL in an active population of HZ patients.

## Methods

### Study design

This is a pooled analysis of the MASTER studies conducted in 8 countries, which shared the same design and were conducted using similar methodology [16–19]. The objectives of the MASTER study were to measure HZ-related burden of illness, HRQoL, health care resource utilisation (HCRU), and out of pocket costs. Among the 1477 patients enrolled overall, 428 were engaged in full or part time work (active population) and were, thus, included in the current analysis, with the following geographic distribution: Latin America (*n* = 128), consisting of Argentina (*n* = 37), Brazil (*n* = 36), Costa Rica (*n* = 6), and Mexico (*n* = 49); North America, consisting of Canada (*n* = 160); and Asia (*n* = 140), consisting of Korea (*n* = 45), Taiwan (*n* = 49), and Thailand (*n* = 46).

### Patient population

Patients eligible for cohort inclusion were either male or female patients ≥ 50 years of age with HZ rash or residual HZ-associated pain, defined as pain persisting subsequent to rash healing. In addition, in order to be included in this analysis, patients had to belong to the active population (actively employed in full or part time work). Incident cases were defined as patients recruited from the offices of general practitioners or specialists for a current HZ episode (rash onset or start of pain) with a duration of ≤ 7 days; prevalent cases were defined as patients enrolled experiencing a HZ episode which had lasted longer than 7 days, with the onset of rash recorded in medical records. Key exclusion criteria were the presence of any medical condition that, in the opinion of the treating physician, could interfere with the evaluations required by the study, and patient and/or family member or primary caregiver refusal to sign informed consent.

### Treatment and follow-up

In accordance with the observational nature of the studies, any treatment of the HZ-episode was based on the judgement of the treating physician. After the baseline assessment at Day 0, patients were followed for a maximum of 6 months. Regardless of the phase of disease at the time of enrolment, 9 follow-up assessments were recommended at Days 7, 14, 21, 30, 60, 90, 120, 150 and 180, to a total of 10 visits, with the exception of Korea and Taiwan, in which prevalent cases were assessed at every month after the baseline visit, for a total of 7 visits overall. The baseline (Day 0) assessment was conducted at the physician’s office, and follow-up assessments were conducted though self-administered questionnaires. At baseline, information regarding the patient’s immune status, pain-related medical history, demographics, current medications, and characteristics of the current HZ-episode, was collected. The outcome measures described in the following section were evaluated at each patient assessment.

### Pooled analysis outcome measures

#### Work productivity and work time loss

Work productivity of patients and/or caregivers was measured at every patient assessment irrespective of the presence of HZ rash and/or pain using a simple descriptive, self-administered, standardized questionnaire, the Work and Productivity Questionnaire (WPQ) (Additional file 1). The WPQ evaluated the number of times work was missed (full and half days), with Work Time Loss defined as missing ≥ 1 partial or full work day. The principal causes of absences (health care visits, pain, discomfort, lack of concentration, visible rash, or medication side effects), and whether or not extended sick leave, disability, or use of vacation time was required, was also assessed. In addition, patients were asked to rate their productivity (effectiveness) at work during their shingles episode on a scale of 0–100%.

#### Health related quality of life

HRQoL was captured using the Euro-QoL (EQ-5D) questionnaire [20], a generic health status instrument which evaluates quality of life based on the measurement of five dimensions: mobility, self-care, usual activities, pain/discomfort and anxiety/depression. A preference based scale, the EQ-5D assesses each dimension with three levels of severity; 1 (no problems), 2 (some problems), and 3 (maximum problems). Each score can then be weighted to adjust for population-specific preferences in health-care states. For the purposes of this analysis, UK weights, the most validated weights, were used to convert individual health dimensions scores to a single EQ-5D summary score, with EQ-5D summary scores closest to 1 indicative of a better quality of life. The VAS component of the EQ-5D questionnaire (EQ-5D VAS) also records the patient’s self-rated health on a horizontal scale, ranging from “worst imaginable health state” to “best imaginable health state”. At the baseline visit (Day 0), each patient was required to complete two EQ-5D questionnaires, one to assess their usual quality of life prior to HZ onset, and another to assess their current state of health during the current HZ episode.

#### HZ-associated pain

HZ-associated pain was evaluated with the Zoster Brief Pain Inventory (ZBPI) questionnaire [21] and the Initial Zoster Impact Questionnaire (IZIQ). The ZBPI is a 9 question HZ-specific questionnaire which evaluates two components of pain, intensity and interference, on an 11 point Likert scale. More specifically, the ZBPI measures the presence and location of pain, the severity of the worst, least, and average pain in the last 24 h, current pain intensity, use of medications, use of relief medications, and the interference of pain on general activity, mood, walking ability, normal work, relations with other people, sleep and enjoyment of life. The IZIQ, completed only at baseline, was used to complement the ZBPI, which was completed at all Study Visits, by assessing pain prior to study enrolment [16–19].

### Statistical analysis

Descriptive statistics were produced for all relevant variables, including the mean and standard deviation for continuous scale variables, and frequency distributions for categorical variables. In addition to the total active population, all analyses were stratified by Work Time Loss Category (Work Time Loss (WTL) versus No Work Time Loss (NWTL)). Significance was determined *a priori* at *p* < 0.05, and a statistical trend was defined at *p* < 0.150.

For assessment of the correlation between HRQoL (EQ-5D item scores: domain scores, overall summary score and VAS) and percentage of work effectiveness, the Pearson product–moment correlation was used. To identify predictors of HRQoL, mixed models with repeated measures were used, where individual EQ-5D scores throughout the follow-up period were the dependent variable, and the following covariates were considered: Work Time Loss Category (WTL versus NWTL), age at rash onset, gender, impaired immune status, presence of HZ complications, severity of rash at baseline (number of HZ lesions), worst pain score at baseline (based on the ZBPI “worst pain in the last 24 h” score), employment status (full-time versus part time), geographic region, and country income. Impaired immune status was defined as the use of high dose oral corticosteroids, invasive cancers (with the exception of CIS and non-melanoma skin cancer), HIV infection/ AIDS, immune deficiency, receipt of chemotherapy for cancer, prior or concurrent immunosuppressive therapy, or receipt of immunosuppressive therapy for organ transplant. Country income categories were determined according to the 2016 World Bank Income categories: upper-middle-income economies were defined as those with a gross national income (GNI) per capita of more than $4126 but less than $12,735; high-income economies were defined as those with a GNI per capita of $12,735 or more [22].

## Results

### Baseline socio-demographic and disease characteristics

Baseline socio-demographic and disease characteristics are presented in Tables 1 and 2, respectively, and are presented overall, as well as stratified by whether or not the patient experienced work time loss (WTL group vs. NWTL group). Of the 428 patients included in the analysis, 247 (57.7%) reported losing work time due to their current HZ episode, with 147 patients (34.3%), reporting no work time loss. Information on work time loss was not available for 34 patients (7.9%) (Table 1). Overall, the mean (SD) age of rash onset was 58.9 (7.2) years, with over 60% of patients between the ages of 50–59. No significant differences were reported in baseline socio-demographic characteristics, with the exception of geographic region, where the proportion of patients from Asian, Latin and North American countries differed across both the WTL and NWTL groups (*p =* 0.010) (Table 1). The majority of patients (*n* = 336; 78.5%) were employed full time, and 92 (21.5%) were part-time workers. Regarding work effectiveness, mean (SD) percent work effectiveness of patients in the WTL group was significantly lower compared to the NWTL group at baseline [50.3 (31.6) vs. 71.4 (27.8); *p* < 0.001], with a significantly greater proportion of patients in the WTL group reporting a decrease in work effectiveness (89.1% in the WTL group versus, 66.0% in the NWTL group; *p <* 0.001) (Table 1). Overall, mean (SD) work time loss reported was 9.1 (15.6) days.

**Table 1:** Baseline socio-demographic characteristics overall and by Work Time Loss Category

Variable	Work time loss category		Overall^b^	*p*-value
	Work time loss^a^	No work time loss		
Total n, %	247 (57.7)	147 (34.3)	428	-
Age at rash onset, years, mean (SD)	59.0 (7.4)	58.6 (7.1)	58.9 (7. 2)	0.674
Gender, female, n (%)	126 (51.0)	71 (48.3)	217 (50.7)	0.602
Age category at rash onset, years, n (%)				
50–59	148 (59.9)	95 (64.6)	263 (61.4)	
60–69	79 (32.0)	39 (26.5)	129 (30.1)	0.520
≥ 70	20 (8.1)	13 (8.8)	35 (8.2)	
Not Available	0 (0.0)	0 (0.0)	1 (0.2)	
Education, n (%)				
Primary school or less	54 (21.9)	25 (17.0)	86 (20.1)	
High school	77 (31.2)	51 (34.7)	139 (32.5)	0.485
College/University	114 (46.2)	69 (46.9)	199 (46.5)	
Not Available	2 (0.8)	2 (1.4)	4 (0.9)	
Geographic region, n (%)^c^				
Asia	77 (31.2)	52 (35.4)	140 (32.7)	
Latin America	91 (36.8)	33 (22.4)	128 (29.9)	0.010
North America	79 (32.0)	62 (42.2)	160 (37.4)	
Country category (income)^d^				
Upper-Middle	94 (38.1)	42 (28.6)	146 (34.1)	
High	93 (37.7)	64 (43.5)	171 (40.0)	0.079
Not Available	60 (24.3)	41 (27.9)	111 (25.9)	
Employment status, n (%)				
Full time	200 (81.0)	112 (76.2)	336 (78.5)	
Part time	47 (19.0)	35 (23.8)	92 (21.5)	0.258
Number of hours work overall/week				
*n*	246	146	411	
Mean (SD)	39.3 (16.0)	38.3 (17.6)	38.8 (16.5)	0.395
Number of hours part time work/week				
*n*	47	34	89	
Mean (SD)	23.3 (11.5)	22.9 (11.7)	23.6 (12.2)	0.939
Number of hours full time work /week				
*n*	199	112	322	
Mean (SD)	43.1 (14.4)	43.0 (16.4)	43.0 (15.0)	0.703
Total number persons/household				
*n*	243	145	421	
Mean (SD)	2.8 (1.6)	3.0 (1.9)	2.8 (1.7)	0.420
Type of household, n (%)				
Apartment	76 (30.8)	48 (32.7)	134 (31.3)	
House	170 (68.8)	97 (66.0)	291 (68.0)	0.838
Other	1 (0.4)	1 (0.7)	2 (0.5)	
Not Available	0 (0.0)	1 (0.7)	1 (0.2)	
Work effectiveness, mean percent, (SD)	50.3 (31.6)	71.4 (27.8)	56.0 (32.0)	<0.001
Work effectiveness category, n (%)				
100%	20 (8.1)	41 (27.9)	67 (15.7)	
50–90%	122 (49.4)	71 (48.3)	206 (48.1)	<0.001
10–40%	61 (24.7)	19 (13.0)	87 (20.3)	
0%	37 (15.0)	7 (4.8)	10 (2.3)	
Not Available	7 (2.8)	9 (6.1)	18 (4.2)	

^a^Patients with Work Time Loss were defined as those who reported missing work due to their shingles episode (entire day or part of a day) at baseline, as assessed by the WPQ

^b^34 patients did not have information on Work Time Loss Category

^c^Asia = Korea, Taiwan, Thailand; Latin America = Argentina, Brazil, Costa Rica, Mexico; North America = Canada

^d^Country income classifications are based on the 2016 World Bank economic definitions [22]. High Income= Canada, South Korea, Taiwan, Argentina; Upper Middle Income= Brazil, Costa Rica, Mexico, Thailand

**Table 2:** Baseline disease parameters overall and by Work Time Loss Category

Variable	Work Time Loss Category		Overall^b^	*p*-value
	Work Time Loss^a^	No Work Time Loss		
Total n, %	247 (57.7)	147 (34.3)	428	-
Time from HZ onset, days				
*n*	247	147	427	
Mean (SD)	124.2 (458.3)	79.6 (284.2)	128.6 (476.6)	<0.001
Time from HZ onset - categorical, n (%)				
Incident	74 (30.0)	65 (44.2)	154 (36.0)	
Prevalent	173 (70.0)	82 (55.8)	273 (63.8)	0.004
Not available	0 (0.0)	0 (0.0)	1 (0.2)	
Pain before rash appearance, n (%)				
Yes	156 (63.2)	89 (60.5)	260 (60.7)	
No	79 (32.0)	52 (35.4)	146 (34.1)	0.520
Not available	12 (4.9)	6 (4.1)	22 (5.1)	
Average pain score before rash appearance^c^				
*n*	155	88	258	
Mean (SD)	4.9 (2.6)	4.4 (2.5)	4.7 (2.6)	0.148
Worst pain before rash appearance^c^				
*n*	154	86	255	
Mean (SD)	6.4 (2.6)	5.8 (2.5)	6.2 (2.6)	0.057
Pain since rash appearance, n (%)				
Yes	222 (89.9)	130 (88.4)	376 (87.9)	
No	14 (5.7)	12 (8.2)	33 (7.7)	0.349
Not available	11 (4.5)	5 (3.4)	19 (4.4)	
Average pain since rash appearance^c^				
*n*	222	129	375	
Mean (SD)	5.6 (2.3)	4.7 (2.2)	5.3 (2.3)	0.002
Worst pain since rash appearance^c^				
*n*	221	128	373	
Mean (SD)	7.6 (2.3)	6.5 (2.5)	7.2 (2.5)	<0.001
Pain in the last 24 hs				
Yes	224 (90.7)	126 (85.7)	380 (88.8)	
No	23 (9.3)	20 (13.6)	47 (11.0)	0.178
Not available	0 (0.0)	1 (0.7)	1 (0.2)	
Average pain in last 24 h^c^				
*n*	224	126	380	
Mean (SD)	4.5 (2.5)	4.0 (2.3)	4.3 (2.4)	0.062
Worst pain in last 24 h^c^				
*n*	220	124	372	
Mean (SD)	6.2 (2.6)	5.4 (2.6)	5.9 (2.6)	0.004
Worst pain score category, n (%)^d^				
Mild	43 (17.4)	35 (23.8)	83 (19.4)	
Moderate	91 (36.8)	55 (37.4)	161 (37.6)	0.041
Severe	86 (34.8)	33 (22.4)	127 (29.7)	
Not available	27 (10.9)	24 (16.3)	57 (13.3)	
Severity of rash (number of lesions), n (%)				
No rash	56 (22.7)	18 (12.2)	84 (19.6)	
Mild (1–20)	107 (43.3)	86 (58.5)	207 (48.4)	
Moderate (21–50)	49 (19.8)	24 (16.3)	77 (18.0)	0.014
Severe (>50)	35 (14.2)	18 (12.2)	59 (13.8)	
Not available	0 (0.0)	1 (0.7)	1 (0.2)	
Impaired immune status^e^				
Yes	18 (7.3)	9 (6.1)	29 (6.8)	
No	229 (92.7)	138 (93.9)	399 (93.2)	0.658
Presence of HZ complication				
Yes	80 (32.4)	40 (27.2)	136 (31.8)	
No	166 (67.2)	107 (72.8)	291 (68.0)	0.269
Not available	1 (0.4)	0 (0.0)	1 (0.2)	
Medication for HZ				
Yes	23 (9.3)	24 (16.3)	51 (11.9)	
No	224 (90.7)	123 (83.7)	377 (88.1)	0.038

*SD* standard deviation, *HZ* Herpes Zoster, *CIS* carcinoma in situ, *HIV* human immunodeficiency virus, *AIDS* acquired immune deficiency syndrome

^a^Patients with Work Time Loss were defined as those who reported missing work due to their shingles episode (entire day or part of a day) at baseline, as assessed by the WPQ

^b^34 patients did not have information on Work Time Loss Category

^c^Measured on an 11 point Likert scale which ranges from “no pain” (0) to “pain as bad as you can imagine” (11)

^d^Worst pain score categories are based on the ZPBI “worst pain in the last 24 h” scores: mild worst pain = ZBPI scores 0- ≤ 3; moderate worst pain = ZBPI scores 4- ≤ 7; severe worst pain = ZBPI score ≥8

^e^Defined as: use of high dose oral corticosteroids, invasive cancers (with the exception of CIS and non-melanoma skin cancer), HIV infection/ AIDS, immune deficiency, chemotherapy for cancer, prior or concurrent immunosuppressive therapy, and therapy for organ transplant

At baseline, rash was predominately absent or mild in severity (68.0%, *n* = 291), with significant differences observed between the WTL and NWTL groups (*p* = 0.014) in terms of the proportion of patients reporting no rash (22.7% WTL vs. 12.2% NWTL), mild rash (43.3% WTL vs. 58.5% NWTL), moderate rash (19.8% WTL vs. 16.3% NWTL), and severe rash (14.2% WTL vs. 12.2% NWTL), and more patients in the NWTL group administered medication for their HZ episode (9.3% WTL vs. 16.3% NWTL; *p* = 0.038) (Table 2). Time from HZ onset was also significantly longer in the WTL group compared to the NWTL group [124.2 (458.3) vs. 79.6 (284.2) days; *p* < 0.001].

Generally, at baseline, patients in the WTL group reported more severe disease parameters, with significant differences found for worst pain in the last 24 h [6.2 (2.6) WTL vs. 5.4 (2.6) NWTL; *p* = 0.004] and average and worst pain since rash experience [5.6 (2.3) WTL vs. 4.7 (2.2) NWTL, *p =*0.002; 7.6 (2.3) WTL vs. 6.5 (2.5) NWTL; *p* < 0.001, respectively]; although differences in the proportion of patients experiencing post-rash pain were not significant (*p* = 0.349) (Table 2). In addition, a significant difference in worst pain (based on the ZBPI “worst pain in the last 24 h” score) was found between Work Time Loss Categories, with an overall greater proportion of patients in the WTL group reporting severe worst pain compared to the NWTL group (34.8% vs. 22.4%; *p* = 0.041) (Table 2). Duration of pain, i.e. from baseline to resolution, was also significantly higher in the WTL group compared to the NWTL group [89.9 (193.7) vs. 53.4 (51.4) days; *p* < 0.001]. No significant differences were found between groups with regards to prodromal pain. Moreover, at baseline, patients in the WTL group had significantly lower (*p* < 0.001) overall HRQoL (EQ-5D summary score), greater problems with self-care and usual activities, and experienced higher pain/discomfort, when compared to patients in the NWTL group (Table 3). Pooled across all visits, significantly lower (*p* < 0.001) scores were seen in the WTL group for both overall HRQoL (EQ-5D summary score and VAS) and individual EQ-5D dimensions (Table 3).

**Table 3:** EQ-5D items and scores at baseline and pooled over time

Variable	Baseline				Pooled visits^c^			
	Work time loss^a^	No work time loss	Overall^b^	*p*-value	Work time loss^a^	No work time loss	Overall^b^	*p*-value
Total n^d^	247	147	428	-	786	2148	3738	
Mobility, n (%)								
I have no problems in walking	174 (70.4)	117 (79.6)	318 (74.3)		554 (71.9)	1876 (89.7)	3019 (80.9)	
I have some problems in walking	65 (26.3)	29 (19.7)	99 (23.1)		202 (26.2)	184 (8.8)	553 (14.8)	
I am confined to a bed	5 (2.0)	0 (0.0)	5 (1.2)	0.051	8 (1.0)	4 (0.2)	13 (0.3)	<0.001
Not available	3 (1.2)	1 (0.7)	6 (1.4)		7 (0.9)	27 (1.3)	147 (3.9)	
Self-care, n (%)								
I have no problems in self-care	191 (77.3)	132 (89.8)	351 (82.0)		631 (81.8)	1988 (95.1)	3260 (87.4)	
I have some problems washing and dressing	49 (19.8)	13 (8.8)	66 (15.4)		125 (16.2)	71 (3.4)	310 (8.3)	
I am unable to wash and dress myself	4 (1.6)	1 (0.7)	5 (1.2)	0.005	8 (1.0)	5 (0.2)	15 (0.4)	<0.001
Not available	3 (1.2)	1 (0.7)	6 (1.4)		7 (0.9)	27 (1.3)	147 (3.9)	
Usual activities, n (%)								
I have no problems with performing usual activities	124 (50.2)	102 (69.4)	249 (58.2)		375 (48.6)	1773 (84.8)	2695 (72.2)	
I have some problems performing usual activities	103 (41.7)	41 (27.9)	153 (35.7)		357 (46.3)	283 (13.5)	821 (22.0)	
I am unable to perform usual activities	17 (6.9)	3 (2.0)	20 (4.7)	0.001	32 (4.2)	8 (0.4)	69 (1.8)	<0.001
Not available	3 (1.2)	1 (0.7)	6 (1.4)		7 (0.9)	27 (1.3)	147 (3.9)	
Pain/Discomfort, n (%)								
I have no pain or discomfort	54 (21.9)	45 (30.6)	110 (25.7)		197 (25.6)	1379 (65.9)	1955 (52.4)	
I have moderate pain or discomfort	142 (57.5)	84 (57.1)	245 (57.2)		459 (59.5)	655 (31.3)	1428 (38.3)	
I am extreme pain or discomfort	48 (19.4)	17 (11.6)	67 (15.7)	0.044	108 (14.0)	30 (1.4)	202 (5.4)	<0.001
Not available	3 (1.2)	1 (0.7)	6 (1.4)		7 (0.9)	27 (1.3)	147 (3.9)	
Anxiety/Depression, n (%)								
I am not anxious or depressed	122 (49.4)	88 (59.9)	230 (53.7)		381 (49.4)	1672 (80.0)	512 (58.9)	
I am moderately anxious or depressed	96 (38.9)	49 (33.3)	156 (36.4)		325 (42.2)	367 (17.6)	910 (24.4)	
I am extremely anxious or depressed	26 (10.5)	9 (6.1)	36 (8.4)	0.097	58 (7.5)	25 (1.2)	110 (2.9)	<0.001
Not available	3 (1.2)	1 (0.7)	27 (3.1)		7 (0.9)	27 (1.3)	147 (3.9)	
EQ-5D summary scores based on UK weight								
*n*	244	146	422		764	2064	3585	
Mean (SD)	0.6 (0.4)	0.7 (0.3)	0.6 (0.3)	0.001	0.6 (0.3)	0.9 (0.2)	0.8 (0.3)	<0.001
VAS, mm								
*n*	244	145	421		763	2059	3572	
Mean (SD)	66.7 (23.7)	71.1 (20.5)	68.5 (22.4)	0.106	68.1 (22.6)	86.5 (16.6)	80.3 (21.0)	<0.001

*SD* standard deviation, *VAS* visual analogue scale, *WPQ* Work and Productivity Questionnaire

^a^Patients with Work Time Loss were defined as those who reported missing work due to their shingles episode (entire day or part of a day) at baseline, or at any of the follow-up visits (Visits 2–10) as assessed by the WPQ

^b^34 patients did not have information on Work Time Loss Category

^c^Visits 1–10

^d^Total n’s are based on cumulative visits

### Correlation analyses

Table 4 presents the correlation between percentage of work effectiveness and all EQ-5D items (dimension scores, summary score, and the VAS), overall, and by Work Time Loss Category. All correlation coefficients (*r)* reported were found to be statistically significant (*p <* 0.001). Overall, percent work effectiveness was negatively correlated with all 5 dimension scores, whereas the EQ-5D summary score and VAS were positively correlated with percentage of work effectiveness (*r* = 0.427 and 0.490, respectively) suggesting that higher work productivity is associated with improved HRQoL. Similar results were observed for the WTL group, although a stronger correlation was observed for percent work effectiveness per the NWTL group versus the WTL group. This was evident in particular with regards to the EQ-5D overall summary score (NWTL: *r =* 0.402 vs. WTL *r =* 0.209) and the VAS (NWTL: *r =* 0.511 vs. WTL: *r* = 0.260).

**Table 4:** Correlation between EQ-5D item scores and percentage of work effectiveness overall and by Work Time Loss Category

Variable	Work Time Loss Category	EQ-5D item scores	Correlation coefficient ^b^	*P*-value
Percent work effectiveness^c^	Overall	Mobility	−0.265	<0.001
		Self-Care	−0.243	<0.001
		Usual activities	−0.401	<0.001
		Pain/Discomfort	−0.396	<0.001
		Anxiety/Depression	−0.331	<0.001
		EQ-5D summary scores based on UK weight	0.427	<0.001
		EQ-5D VAS	0.490	<0.001
		Mobility	−0.238	<0.001
		Self-Care	−0.158	<0.001
		Usual activities	−0.323	<0.001
	No Work Time Loss	Pain/Discomfort	−0.356	<0.001
		Anxiety/Depression	−0.329	<0.001
		EQ-5D summary scores based on UK weight	0.402	<0.001
		EQ-5D VAS	0.511	<0.001
		Mobility	−0.148	<0.001
		Self-Care	−0.162	<0.001
		Usual activities	−0.245	<0.001
	Work Time Loss^a^	Pain/Discomfort	−0.161	<0.001
		Anxiety/Depression	−0.140	<0.001
		EQ-5D summary scores based on UK weight	0.209	<0.001
		EQ-5D VAS	0.260	<0.001

*WPQ* Work and Productivity Questionnaire, *VAS* visual analogue scale

^a^Patients with Work Time Loss were defined as those who reported missing work due to their shingles episode (entire day or part of a day) at baseline, or at any of the follow-up visits (Visits 2–10) as assessed by the WPQ

^b^Correlation coefficient was calculated based on Pearson’s correlation measure

^c^Pooled over time (Visits 1–10)

### Multivariate analyses

Upon adjusting for Work Time Loss Category, individual predictors of quality of life (both the EQ-5D summary score and the VAS) were time since HZ onset, worst pain score, severity of rash at baseline, geographic region, and country income (Table 5). Presence of HZ complications was also identified as a potential predictor of the VAS. Significant independent predictors of EQ-5D overall summary score identified in the saturated multivariate model are presented in Table 6. Work time loss was identified as a significant negative predictor of HRQoL with regards to both the EQ-5D overall summary score and the VAS (*p* < 0.001), as was moderate/severe worst pain score compare to mild pain (*p* < 0.001). Increased severity of rash was associated with significantly higher EQ-5D summary score, (*p* = 0.042 for mild vs. no rash; *p* = 0.206 for moderate vs. no rash; *p* = 0.017 for severe vs. no rash), whereas country income (high vs. upper middle income levels) and presence of HZ complications, were both significant negative predictors (*p* = 0.003, and *p* = 0.007) of the VAS.

**Table 5:** Repeated measures mixed model analysis assessing individual predictors of the EQ-5D summary score and EQ-5D VAS

	EQ-5D Item							
	EQ-5D overall summary score				EQ-5D VAS			
Predictor	Estimate^a^	SD	95% CI for estimate	*p*-value	Estimate^a^	SD	95% CI for estimate	*p*-value
Work Time Loss Category								
Work Time Loss^b^ vs. No Work Time Loss	−0.234	0.010	−0.254, −0.215	<0.001	−16.92	0.755	−18.40, −15.44	<0.001
Time from HZ onset - categorical								
Prevalent vs. incident	−0.049	0.014	−0.076, −0.022	<0.001	−3.811	1.339	−6.442, −1.181	0.005
Age category at rash onset, years^c^								
60–69 vs. 50–59	−0.002	0.015	−0.032, 0.028	0.898	−1.562	1.465	−4.441, 1.318	0.287
≥ 70 vs. 50–59	−0.042	0.026	−0.092, 0.008	0.100	−2.785	2.472	−7.643, 2.073	0.260
Worst Pain Category^c,d^								
Moderate vs. mild	−0.084	0.013	−0.111, −0.058	<0.001	−8.842	1.075	−10.95, −6.732	<0.001
Severe vs. mild	−0.287	0.018	−0.323, −0.251	<0.001	−14.08	1.447	−16.92, −11.24	<0.001
Severity of rash (number of lesions)^c^								
Mild (1–20) vs.no rash	0.109	0.017	0.074, 0.143	<0.001	10.646	1.707	7.292, 14.000	<0.001
Moderate (21–50) vs. no rash	0.100	0.021	0.058, 0.141	<0.001	8.892	2.064	4.837, 12.947	<0.001
Severe (>50) vs. no rash	0.095	0.024	0.049, 0.142	<0.001	9.684	2.278	5.207, 14.160	<0.001
Gender^c^								
Male vs. female	−0.005	0.013	−0.032, 0.021	0.691	0.833	1.295	−1.711, 3.377	0.520
Impaired immune status^c, e^								
Yes vs. no	−0.034	0.025	−0.084, 0.016	0.181	−1.876	2.472	−6.734, 2.983	0.448
Presence of complications from HZ^c^								
Yes vs. no	−0.012	0.014	−0.040, 0.016	0.400	−5.016	1.370	−7.707, −2.324	<0.001
Employment status^c^								
Full time vs. part time	0.008	0.016	−0.024, 0.040	0.612	−1.919	1.574	−5.011, 1.173	0.223
Geographic region^c,f^								
Latin America vs. Asia	0.030	0.017	−0.004, 0.064	0.191	3.245	1.628	0.047, 6.443	0.098
North America vs. Asia	−0.043	0.017	−0.076, −0.010	0.011	−5.146	1.583	−8.257, −2.035	0.001
Country category (income)^c, g^								
High vs. Upper Middle	−0.052	0.014	−0.079, −0.026	<0.001	−6.616	1.290	−9.151, −4.081	<0.001

*SD* standard deviation, *CI* confidence interval, *HZ* Herpes Zoster, *WPQ* Work and Productivity Questionnaire, *ZBPI* Zoster Brief Pain Inventory, *VAS* visual analogue scale, *CIS* carcinoma in situ, *HIV* human immunodeficiency virus, *AIDS* acquired immune deficiency syndrome

^a^The estimate is the relative effect of the predictor on EQ-5D scores compared to the reference group

^b^Patients with Work Time Loss were defined as those who reported missing work due to their shingles episode (entire day or part of a day) at baseline, or at any of the follow-up visits (Visits 2–10) as assessed by the WPQ

^c^Predictors were adjusted by Work Time Loss Category

^d^Worst pain score categories are based on the ZBPI “worst pain in the last 24 h” scores: mild worst pain = ZBPI scores 0- ≤ 3; moderate worst pain = ZBPI scores 4- ≤ 7; severe worst pain = ZBPI score ≥8

^e^ Defined as: use of high dose oral corticosteroids, invasive cancers (with the exception of CIS and non-melanoma skin cancer), HIV infection/ AIDS, immune deficiency, chemotherapy for cancer, prior or concurrent immunosuppressive therapy, and therapy for organ transplant

^f^Asia = Taiwan, Thailand, South Korea; Latin America = Argentina, Brazil, Costa Rica; Mexico; North America = Canada

^g^Country income classifications are based on the 2016 World Bank economic definitions [22]. High Income= Canada, South Korea, Taiwan, Argentina; Upper Middle Income= Brazil, Costa Rica, Mexico, Thailand

**Table 6:** Saturated multivariate repeated measures mixed model assessing independent predictors of the EQ-5D summary score and EQ-5D VAS

	EQ-5D Item							
	EQ-5D overall summary scores				EQ-5D VAS			
Predictor	Estimate^a^	SD	95% CI for estimate	*p*-value	Estimate^a^	SD	95% CI for estimate	*p*-value
Work Time Loss Category								
Work Time Loss^b^ vs. No Work Time Loss	−0.102	0.014	−0.129, −0.074	<0.001	−6.511	1.109	−8.687, −4.336	<0.001
Time from HZ onset - categorical								
Prevalent vs. incident	−0.016	0.019	−0.052, 0.020	0.387	−0.871	1.754	−4.320, 2.578	0.620
Worst Pain Category^c^								
Moderate vs. mild	−0.083	0.014	−0.109, −0.056	<0.001	−8.761	1.079	−10.88, −6.643	<0.001
Severe vs. mild	−0.290	0.018	−0.326, −0.254	<0.001	−14.40	1.445	−17.23, −11.56	<0.001
Severity of rash (number of lesions)								
Mild (1–20) vs. no rash	0.044	0.021	0.002, 0.086	0.042	4.150	2.128	0.035, 8.334	0.052
Moderate (21–50) vs. no rash	0.035	0.028	−0.019, 0.090	0.206	3.755	2.667	−1.489, 8.999	0.160
Severe (>50) vs. no rash	0.070	0.029	0.012, 0.128	0.017	4.621	2.865	−1.014, 10.255	0.108
Country category (income)^d^								
High vs. Upper Middle	−0.027	0.033	−0.091, 0.038	0.413	−8.532	2.902	−14.24, −2.828	0.003
Geographic region^e^								
Latin America vs. Asia	0.021	0.032	−0.042, 0.083	0.513	−2.793	2.839	−8.374, 2.788	0.326
North America vs. Asia	0.005	0.024	−0.043, 0.052	0.851	1.428	2.253	−3.001, 5.856	0.527
Presence of complications of HZ								
Yes vs. no	-	-	-	-	−4.509	1.654	−7.761, −1.257	0.007

*SD* standard deviation, *CI* confidence interval, *VAS* visual analogue scale, *WPQ* Work and Productivity Questionnaire

^a^The estimate is the relative effect of the predictor on EQ-5D scores compared to the reference group

^b^ Patients with Work Time Loss were defined as those who reported missing work due to their shingles episode (entire day or part of a day) at baseline, or at any of the follow-up visits (Visits 2–10) as assessed by the WPQ

^c^ Worst pain categories are based on the ZBPI “worst pain in the last 24 h” scores: mild worst pain = ZBPI scores 0- ≤ 3; moderate worst pain = ZBPI scores 4- ≤ 7; severe worst pain = ZBPI score ≥8

^d^ Country income classifications are based on the 2016 World Bank economic definitions [22]. High Income= Canada, South Korea, Taiwan, Argentina; Upper Middle Income= Brazil, Costa Rica, Mexico, Thailand

^e^ Asia = Taiwan, Thailand, South Korea; Latin America = Argentina, Brazil, Costa Rica; Mexico; North America = Canada

## Discussion

As reported previously, the results of this analysis demonstrate that shingles has a negative impact on the work and productive life of individuals [9–11]. Individuals experiencing work time loss reported lower health utility scores, at baseline and overall, than their non-work time loss counterparts, with work time loss identified as an independent negative predictor of both the EQ-5D summary scores and the VAS (*p* < 0.001). Decrease in work effectiveness was also negatively associated with quality of life overall, and in both the WTL and NWTL groups.

Additional predictors of quality of life identified were worst pain score, the presence of HZ complications, and country income (both complications and county income predictors of the EQ-5D VAS only). Although severity of rash was identified as a significant predictor of quality of life, the direction of the prediction is of interest, with more severe rash associated with improved HRQoL. This may be explained by the fact that, due to the inclusion of prevalent cases, there may not be a temporal association between rash assessment and disease onset. Thus, it could be argued that patients with no rash were those for whom rash healing had occurred, and that consequently, these patients experienced an overall longer time elapsed since disease onset. As it has been documented that prolonged pain of HZ has a significant effect on quality of life [6, 19], the residual post-rash pain experienced by these patients may have resulted in the reporting of lower health utility scores compared to patients assessed earlier in the course of their HZ episode, when rash manifestation was still evident. In our analysis, we have tried to adjust for the effect of time since HZ onset which was not found to have a significant impact on quality of life; however, it is possible that there may be residual confounding.

Overall 57.7% of patients reported missing work for an average (SD) of 9.1 (15.6) days. Days missed is therefore higher than that reported by Drolet et al. (3.4 days) and Singhal et al. (4 days) [9, 10], however discrepancies in study design may account for these differences: Drolet et al. evaluated HZ patients within 14 days of rash onset, whereas this study’s inclusion of prevalent cases may have resulted in patient recall bias leading to an overestimation of HZ-related work time loss. In addition, at baseline, a higher proportion of patients in the WTL group reported a decrease in work effectiveness, indicating that patients who miss work due to their HZ episode are also less productive, and experience increased presenteeism.

Importantly, our findings show that patients reporting work time loss experience lower quality of life as compared to those not reporting work time loss, independently of differences in disease severity and other potential confounders. This suggests that people, at least some, consider work loss in their valuation of health states. Whether the effect of work time loss on quality of life is due to income loss and/or non-monetary factors such as psychological factors, could not be evaluated. In a review paper by Tilling et al., the proportion of patients who considered monetary losses in health state valuations when explicit instruction was not given was found to vary from 6 to 64% across the studies characterized [23]. In the same paper, significant differences in health care valuations were also observed between groups with and without instruction to consider income. Overall, Tiling et al. concluded that considerable inconsistencies exist between individuals in regards to considering income effects when valuing health states [23]. Due to differences in respondent characteristics, severity of health states values, measurement technique, and country of conduct, these results, as in the Tilling et al. review, were not consistent across all studies, and two were identified in which a majority of respondents did consider income [24, 25]. However, the two studies identified report that even with explicit instruction, the effects on income on health care valuations are disputable: Shiroiwa et al. found no significant differences in utility scores between individuals receiving no instruction regarding income, individuals instructed to consider income reduction, and individuals instructed to assume compensation for lost income [25], and Krol et al. found that explicit instruction on the inclusion of income effects had only some effect on time trade-off (TTO) valuations [24]. This suggests that the effect of lost income on utility scores is multifaceted, as it may involve social aspects such as human relationships and self-fulfillment, rendering the effects of double counting in calculating cost-effectiveness negligible [25].

A limitation of the current analysis is that, due to the inclusion of prevalent cases, time since disease onset varied across patients, and may have led to recall bias in the assessment of work time loss since rash onset and the presence of prodromal pain. Furthermore, disease misclassification cannot be ruled out, as laboratory confirmation of VZV was not protocol-mandated. Finally, it is possible that selection bias towards including more severe HZ cases may have been introduced due to the fact that the patient population comprised of patients who sought out medical attention for their HZ episode.

## Conclusions

The results of this study demonstrate that HZ-related reductions in work effectiveness and work time have a negative effect on the quality of life of actively employed individuals, independently of differences in disease severity and other potential confounders. However, it remains to be determined whether patients consider reductions in income when valuating health care states, as the results of studies assessing the unprompted inclusion of the effects of income have reported conflicting results [25, 26]. In fact, the revised recommendations put forth by the Panel on Cost-Effectiveness in Health and Medicine, in a complete methodological shift, affirm that effects on productivity are unlikely to be captured in the denominator of most preference-based measures, citing inconclusive evidence [15]. Instead, in reference case analyses conducted under the societal perspective, the Panel advocates for the inclusion of these effects in the numerator of the ICER despite uncertainty with respect to the risk of double counting [15].

## Additional file

Additional file 1: Work and Productivity Questionnaire. (PDF 14 kb)

## Abbreviations

AIDS: Acquired immunodeficiency syndrome
CI: Confidence interval
CIS: Carcinoma in situ
EQ-5D: Euro-QoL 5D questionnaire
GNI: Gross national income
HCRU: Health care resource utilization
HIV: Human immunodeficiency virus
HRQoL: Health related quality of life
HZ: Herpes Zoster
ICER: Incremental cost-effectiveness ratio
IZIQ: Initial Zoster Impact Questionnaire
NWTL: No work time loss
QoL: Quality of life
SD: Standard deviation
VAS: Visual analogue scale
VZV: Varicella zoster virus
WPQ: Work and Productivity Questionnaire
WTL: Work time loss
ZBPI: Zoster Brief Pain Inventory
