# Supplementary material for: Association between work time loss and quality of life in patients with Herpes Zoster: a pooled analysis of the MASTER studies
Source: Health Qual Life Outcomes. 2017 Jan 18;15:11. doi: 10.1186/s12955-017-0588-x (PMC5242047; doi:10.1186/s12955-017-0588-x)
Supplement: Additional file 1: — Work and Productivity Questionnaire. (PDF 14 kb) [file 12955_2017_588_MOESM1_ESM.pdf]

MASTER

Monitoring & Assessing Shingles Through Education and Research

WORK AND PRODUCTIVITY QUESTIONNAIRE

These questions are about your work for pay during your shingles episode. Please read and answer each question carefully. If you are not sure how to answer a question, please give the best answer you can.

1. Before your shingles episode, did you work at a paying job?

☐ No > If NO, then skip this questionnaire and go to the Health Care Utilization Resource Use on

☐ Yes > If YES, then please continue

2. Number of times you missed work because of the effects of your shingles, shingles medication, or other treatment for shingles? (If none, please write "0")

an entire day ☐☐ Times

part of a work day ☐☐ Times

3. Please indicate the principle causes of your absences: (please check all that apply)

☐ healthcare visits
☐ too much pain to work
☐ too uncomfortable to work
☐ unable to concentrate at work
☐ visible rash kept you from wanting to go to work
☐ side effects of shingles medication
☐ other (specify): ____________________

4. Please indicate whether you needed to:

a. Take extended sick leave due to your shingles episode?

☐ No

☐ Yes > If YES, for how long? ☐☐☐ days

b. Go on disability due to your shingles episode?

☐ No

☐ Yes > If YES, for how long? ☐☐☐ days

c. Used vacation time to recover from your shingles episode?

☐ No

☐ Yes > If YES, for how long? ☐☐☐ days

5. How would you rate your effectiveness at work during your shingles episode? (check only one box) ☐ N/A (no pain)

0%	10%	20%	30%	40%	50%	60%	70%	80%	90%	100%
Not effective at all										Completely effective (able to work like before shingles began)
